# Effects of Small Deviations in Fiber Orientation on Compressive Characteristics of Plain Concrete Cylinders Confined with FRP Laminates

**DOI:** 10.3390/ma16010261

**Published:** 2022-12-27

**Authors:** Ali Banaeipour, Mohammadreza Tavakkolizadeh, Muhammad Akbar, Zahoor Hussain, Krzysztof Adam Ostrowski, Alireza Bahadori, Mariusz Spyrka

**Affiliations:** 1 Department of Civil Engineering, Ferdowsi University of Mashhad, Mashhad 9177939579, Iran; 2 Institute of Mountain Hazards and Environment, Chinese Academy of Sciences, Chengdu 610041, China; 3 School of Civil Engineering, Zhengzhou University, Zhengzhou 410001, China; 4 Department of Civil Engineering, Sir Syed University of Engineering & Technology, Karachi 75300, Pakistan; 5 Faculty of Civil Engineering, Cracow University of Technology, 24 Warszawska Str., 31-155 Cracow, Poland; 6 Department of Civil Engineering, University of Tehran, Tehran 1417643184, Iran

**Keywords:** confinement, fiber-reinforced polymer, fiber orientation, concrete cylinders, compressive characteristics, finite element modeling

## Abstract

The effectiveness of concrete confinement by fiber-reinforced polymer (FRP) materials is highly influenced by the orientation of fibers in the FRP laminates. In general, acceptable deviation limit from the intended direction is given as 5° in most design guidelines, without solid bases and reasoning. In this paper, a numerical study using finite element modeling was conducted to assess the effects of small deviations in fiber orientation from the hoop direction on compressive behavior of concrete cylinders confined with FRP. Different fiber angles of 0°, 2°, 5°, 8°, 10° and 15° with respect to hoop direction, unconfined concrete compressive strengths of 20, 35 and 50 MPa, FRP thicknesses of 0.2, 0.5 and 1.0 mm and FRP moduli of elasticity of 50 and 200 GPa were considered. The results showed that total dissipated energy ($E_{t}$), ultimate axial strain ($\varepsilon_{cu}^{\prime }$) and compressive strength ($f_{cu}^{\prime }$) exhibited the most reduction with deviation angle. For 5° deviation in fiber orientation, the average reduction in $f_{cu}^{\prime }$, $\varepsilon_{cu}^{\prime }$ and $E_{t}$ were 2.4%, 2.8% and 4.5%, respectively. Furthermore, the calculated allowable limit of deviation in fiber orientation for a 2.5% reduction in $f_{cu}^{\prime }$, $\varepsilon_{cu}^{\prime }$ and $E_{t}$ were 6°, 3° and 2°, respectively, with a 95% confidence.

## 1. Introduction

The need for rapid, safe and efficient repair of concrete structures has grown in recent years [1,2,3]. Repairing, retrofitting and strengthening of damaged concrete columns using fiber-reinforced polymer (FRP) laminates as confinement has become widely accepted in civil infrastructures due to the high tensile strength, light weight and corrosion resistance of these materials [2,3,4,5]. FRP confinement of concrete columns enhances their compressive strength and ductility. Several parameters influence the effectiveness of confinement; among which are compressive strength of plain concrete, modulus of elasticity, FRP thickness and fiber orientation of FRP laminates. To date, however, less attention has been paid to the impact of fiber orientation on behavior of concrete strengthened with FRP laminates [6]. The mechanical behavior of FRP laminates could be maximized by aligning fibers along the optimum orientation. It is important to remember to ensure the proper orientation of the fibers in the laminate when reinforcing each structural element and adjust this direction depending on the stress distribution in the element. In the case of concrete columns subjected to uniaxial compressive loading, it is well established that fibers should be lined up along the hoop (circumferential) direction to restrict the dilation of the concrete core under compression [7,8,9,10,11,12]. In this case, polymer fiber mats with unidirectional reinforcement are most often used for strengthening. Additionally, important mechanical properties of FRP materials, such as strength and modulus of elasticity as shown in fib Bulletin [13], are highly influenced by fiber orientation. Even small variations in fiber angle will result in significant reduction in expected property enhancements [14]. In existing design guidelines, the acceptable limit for the deviation from the intended direction of fiber alignment is given as 5° [15], without any reported reasoning. Therefore, it is important to understand and systematically investigate the influence of deviation in fiber orientation on the behavior of FRP-confined concrete columns under axial loading.

Most studies on FRP-confined concrete columns have focused on the use of fibers in the hoop direction due to the anticipated effect of increasing axial capacity and simplicity. A number of studies reported their findings on FRP-confined concrete specimens with fibers in inclined directions. These studies are mainly focused on the effect of different stacking sequences of cross-ply FRP wraps (e.g., ±45° and ±90°), or different winding angles in the case of concrete-filled FRP tubes (CFFTs), with fiber orientations of more than 15° (not small deviations) with respect to hoop direction [6,7,8,9,10,11,12,16,17,18,19,20,21,22,23,24,25,26,27,28,29,30,31]. No discussions can be found in these studies on how changes in fiber orientation affect the behavior of strengthened concrete. Some studies [6,7,8,9,10,11,12,16,17,18,19,20,21,22,23,24,25,26,27,28,29,30,31], including two numerical investigations [16,17,18,19,20,21,22,23], tested different FRP stacking sequence configurations (such as ±30°, ±45°, ±60°, 0°/±45°, etc.) [6,7,8,9,10,11,12,16,17,18,19,20,21,22,23,24,25,26,27,28,29,30,31], and observed that hoop fibers and inclined cross-ply fibers result in higher strengths and more ductility, respectively. They attributed that to a distinct re-orientation mechanism of fibers in angular cross-ply FRP wraps to dissipate energy, but found these ply mix sequences not mechanically efficient in strength enhancement [10]. Other studies by Micelli et al. [7] examined the effect of various experimental parameters on the confinement effectiveness of FRP made of carbon fibers (CFRP) and glass fibers (GFRP). Elsanadedy et al. [32] demonstrated the insignificant effect of the size of an FRP concrete specimen on the confinement–stress ratio, based on experimental research and non-linear finite element analysis. This discovery will help narrow down the number of samples needed for analysis. However, neither Elsanadedy et al. [32] nor Micelli et al. [7] took into account the effect of small deviations in fiber orientation.

Numerous research studies experimentally investigated the axial compressive behavior of fiber-reinforced polymer tube-confined concrete cylinders in the past two decades [33]. Only a handful of studies investigated the effect of fiber orientation when using unidirectional FRP wraps (not cross-ply wraps). Li et al. [10] and Li [11] studied the effect of fiber orientation on the structural behavior of FRP-confined concrete and found that fiber orientation had a considerable effect on compressive behavior. They observed a reduction in strength and ductility enhancement when fibers are not aligned in the hoop direction, due to possible in-plane shear and transverse tension mechanisms controlling the failure. Vincent and Ozbakkaloglu [6] investigated CFFT specimens with fibers aligned at 15°, 30° and 45° with respect to hoop direction and came to the similar conclusion that the axial compressive behavior is highly sensitive to fiber orientation. It has been proven that the mechanical performance of specimens was optimized when fibers aligned in the hoop direction and a significant reduction in fiber efficiency with deviation from the hoop direction has been observed. It can be concluded from the literature that using stacking sequences consisting of both hoop fibers and inclined cross-ply fibers enhances the ductility of concrete confined with FRP. Moreover, using inclined fibers in the case of single, unidirectional wraps (that is, deviation in fiber orientation with respect to hoop direction) results in lower compressive strength and ductility enhancement, when comparing to straight orientation of fibers.

As discussed before, the majority of existing studies tested a variety of cross-ply wraps with inclined fibers in different stacking sequence configurations and types of mixes. However, in practice, most columns are confined with unidirectional wraps in the hoop direction, and that is where the effects of deviations in fiber orientation and evaluating the current deviation limit of 5° in design codes become important. Moreover, only a comprehensive study of all significant parameters (fiber orientation, unconfined and confined concrete strength, modulus of elasticity of FRP and thickness—number of layers) results in a complete understanding of the effects of deviation in fiber orientation. No study to date has thoroughly investigated the influence of small deviations in fiber orientation, combined with three other significant parameters, on the axial behavior of concrete columns confined with FRP.

Studies were also carried out on FRP strengthening reinforced concrete beams, including those with complex T-beams [34,35]. The change in the behavior of reinforced concrete tees with insufficient shear strength under vertical load as a result of reinforcement with glass fiber-reinforced polymer composites (GFRP) in various configurations was studied by Sadeq A.H. AL-Shalif et al. [34]. In the case of shear, the focus was on aligning the fibers in the direction of the shear force due to the expected effect of increasing the load capacity. The reinforcement of shear-deficient T-beams is not as easy as rectangular beams due to the presence of plates, therefore, the FRP fixing was achieved by both gluing and anchoring [33,34,35]. This study primarily focuses on the effect of small fiber-orientation deviations on the compression characteristics of ordinary concrete cylinders closed with FRP laminates, which cannot be directly related to the described example of beam reinforcement. It is worth noting, however, that the mentioned studies also did not include a comparison of the results obtained for small deviations of the fiber orientation in relation to the assumed values.

As described above, it is rare to analyze the parameters of the FRP composite depending on small deviations of the fiber inclination angle, moreover, the analysis of stress–strain models to predict the strength and strain enhancement ratio of fiber-reinforced polymer tube-confined concrete cylinders under axial compression is also very rare [33]. Therefore, this article is valuable from two perspectives. In their research, Khan Qasim S et al. [33] developed strength and strain enhancement ratio models for circular fiber-reinforced polymer tube-confined concrete under axial compression based on an artificial neural network using experimental test results.

A particularly important issue can be found in the research of Seffo M. et al. [36]. Their experimental results indicated that the strength of the confined concrete cylinder increases in direct proportion to the number of layers of composites, and, moreover, fiber orientation is one of the important parameters that affect strength and ductility of CFRP-confined concrete. This was also noted by Ahmed Sulaimana et al. [37] in their published results of an ongoing experimental investigation examining the effect of fiber orientation and stacking sequence on the behavior of FRP-confined concrete, where it can be seen that the specimens were confined using various CFRP stacking sequences, with fibers oriented at 0°, 90° and 3:45°. It is very important that they included a very small angle in the study. The preliminary results show that parameters such as fiber orientation, stacking sequence and number of confinement layers have a direct impact on the strength, ductility and stress–strain behavior of CFRP-confined concrete [37].

### Research Significance

The key parameters affecting the performance of FRP-confined concrete columns are concrete compressive strength, modulus of elasticity, thickness and fiber orientation of FRP laminates. To date, however, less attention has been paid to fiber orientation. Mechanical properties of FRP materials are highly influenced by fiber orientation, and even small variations in fiber angle will result in major reductions in expected property enhancements. In the current ACI 440.2 design guidelines, the acceptable limit for the deviation from the intended direction of fiber alignment is given as 5°, without any reported reasoning. No study to date has thoroughly investigated the influence of small deviations in fiber orientation, combined with three other key parameters, on the axial behavior of FRP-confined concrete columns. The current study tries to provide a better understanding by systematically investigating the influence of deviation in fiber orientation on the behavior of FRP-confined concrete columns under axial loading.

This paper reports on a numerical study aimed at investigating the effects of small deviations in fiber orientation on important compressive characteristics of concrete columns confined with FRP using finite element analysis (FEA). Firstly, a summary of the finite element (FE) modeling is provided. Then, a validation of the FE modeling by comparing the results with available experimental data is presented. Following this, the results of the parametric study are offered, in which the effects of deviation in fiber orientation with respect to hoop direction on stress–strain behavior, compressive capacity, ultimate deformation and energy dissipation of FRP-confined concrete are discussed. In addition, the loss of ultimate concrete compressive stress and strain along with the reduction in the total dissipated energy corresponding to 5° deviations (allowable value of deviation in most design codes) are presented. Finally, allowable values of deviation in fiber orientation with practical confidences (95% and 99%) for a range of declines in the mentioned properties are suggested.

## 2. Finite Element Modeling

General purpose FEA software Abaqus^®^-Standard (implicit with 100 time steps per numerical specimen) was employed to generate numerical models and simulate the structural response of the concrete columns strengthened with FRP. The output of the generated models was validated against available experimental results.

### 2.1. Geometry, Boundary Conditions, Loading and Failure Criterion

**Figure 1:**
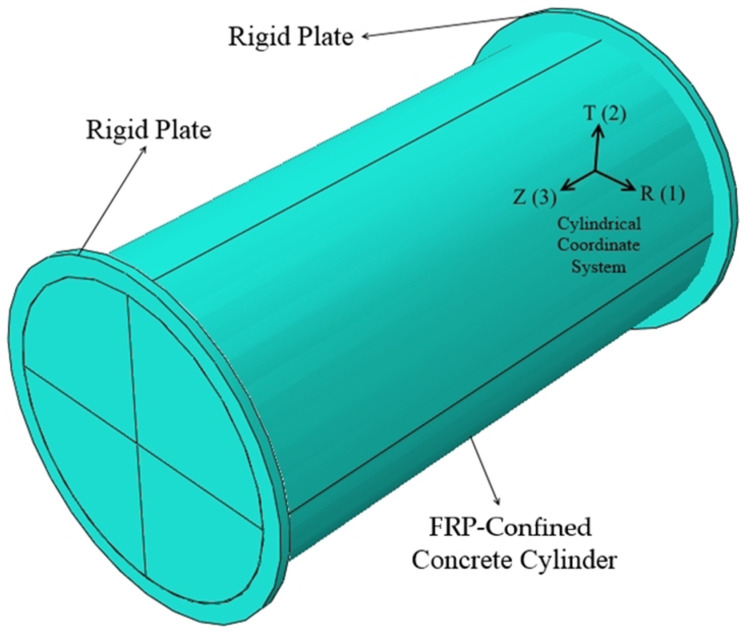
Geometry of the FE model.

The geometry and boundary conditions of the columns are described in this section. Fully wrapped concrete cylinders had dimensions of 152 × 305 mm. Given the geometry of the model, a cylindrical coordinate system was defined and assigned to the specimens. Since the complete stress–strain behavior of the columns was determined, the loading was applied under a displacement control regimen from the top of the samples. In order to apply the axial displacement as loading, the columns were fixed at the top and bottom in all directions, except for the longitudinal direction at the top. Two rigid plates were defined and attached to both ends in order to uniformly distribute the applied displacement. To define the friction between contacting surfaces of the concrete and rigid plates, the Coulomb friction model was used. This model requires the coefficient of friction. For dry interfaces of concrete and steel, the value is suggested as 0.57 [38,39]. It is assumed that there is no relative slip and deboning between the single-ply FRP wrap and concrete, so two parts were tied together. Since many previous researchers reported that the ultimate failure of the confined concrete columns was determined by the rupture of FRP wrap, the failure was controlled by the ultimate tensile strain of the FRP laminate [39,40,41,42,43]. The geometry of the model is illustrated in Figure 1.

### 2.2. Modeling of FRP Wrap

The unidirectional FRP laminate was defined as a deformable shell material with a linear elastic behavior. Shell element “S4R” was selected for FRP laminates. In order to obtain accurate results from the FE modeling, the element size of the FRP wrap and outer surface of the concrete cylinder were assigned as equal to ensure both materials shared the same nodes. Under a plane stress condition, which is the case in a shell element, only the values of $E_{1}$, $E_{2}$, $\nu_{12}$ and $G_{12}$ need to be defined, since unidirectional FRP laminate is considered an “especially orthotropic material” or a “transversely isotropic material”. It should be noted that direction 1 represents fiber direction and direction 2 represents transverse direction. Therefore, for example, $E_{1}$ stands for FRP modulus of elasticity (Young’s modulus) in the fiber direction (direction 1), and $G_{12}$ and $\nu_{12}$ denote FRP shear modulus and FRP Poisson’s ratio in the plane (1,2), respectively.

### 2.3. Modeling of FRP-Confined Concrete

Concrete was considered as an isotropic elastic body and was defined as a deformable material with both elastic and plastic behaviors. Solid element “C3D8R” was selected for concrete. The elastic behavior of concrete is defined by its elastic modulus, $E_{c}$, and Poisson’s ratio, $\nu_{c}$. In this study, $\nu_{c}$ is set to be 0.2, and $E_{c}$ can be obtained from Equation (1) [44]:

(1) $$E_{c}=4730\sqrt{f_{co}^{\prime }\left(\mathrm{MPa}\right)}$$

where $f_{co}^{\prime }$ is the unconfined concrete strength. Additionally, the axial strain at the peak stress of unconfined concrete, $\varepsilon_{co}$, can be obtained from Equation (2) [45], if not available:

(2) $$\varepsilon_{co}=0.000937\sqrt[{4}]{f_{co}^{\prime }\left(\mathrm{MPa}\right)}$$

The plastic behavior of FRP-confined concrete is defined by the linear extended Drucker–Prager (DP) plasticity model. This model has been shown to perform well in modeling the stress–strain behavior of confined concrete [46,47,48]. The accuracy of the model largely depends on the sound evaluation of its parameters that determine the yield criterion, hardening/softening law and flow rule. In order to implement the DP plasticity model, three following key parameters are required: friction angle (φ), flow stress ratio (*K*) and dilation angle (β).

The yield criterion in the linear DP model is defined by the angle of friction, which is assumed to be 54° based on previous studies [47,48]. To ensure that the yield surface remains convex, *K* should lie between 0.78 and 1.00 [49]. In this study, *K* was assumed to be 1.00 for confined concrete. The plastic dilation angle is the major parameter governing the DP flow rule. Jiang et al. [48] established the plastic dilation angle as a function of axial plastic strain ($\varepsilon_{c}^{p}$) and the lateral stiffness ratio (ρ). They subsequently developed two relationships for calculating the plastic dilation angle (Equations (3) and (4)), which were used in this study:

(3) $$\beta =\frac{\beta_{0}+M_{0}\varepsilon_{c}^{p}+\left(0.17\rho^{2}-4.9\rho +1045\right)\beta_{0}\varepsilon_{c}^{p}+\left(0.025e6\rho^{2}-2.52e6\rho +4.27e7\right){\left(\varepsilon_{c}^{p}\right)}^{2}}{1+\left(0.17\rho^{2}-4.9\rho +1045\right)\varepsilon_{c}^{p}+\left(-9767.8\rho +7.3e5\right){\left(\varepsilon_{c}^{p}\right)}^{2}},\rho \leq 35$$

(4) $$\beta =\frac{\beta_{0}+M_{0}\varepsilon_{c}^{p}+\left(0.17\rho^{2}-4.9\rho +1045\right)\beta_{0}\varepsilon_{c}^{p}+(-0.25e6\rho -6.73e6){\left(\varepsilon_{c}^{p}\right)}^{2}}{1+\left(0.17\rho^{2}-4.9\rho +1045\right)\varepsilon_{c}^{p}+\left(6398.7\rho +1.84e5\right){\left(\varepsilon_{c}^{p}\right)}^{2}},\rho >35$$

(5) $$\beta_{0}=37^{{}^{\circ}}\;\&\;M_{0}=157000\;\&\;\rho =\frac{2E_{frp}t_{frp}}{Df_{co}^{\prime }}\;$$

where $E_{frp}$, $t_{frp}$ and *D* are FRP modulus of elasticity in hoop direction, FRP thickness and diameter of concrete cylinder, respectively. $\beta_{0}$ is the initial slope of β, and $M_{0}$ is a constant. Axial plastic strain ($\varepsilon_{c}^{p}$) is a function of lateral stress ($\sigma_{l}$) and is calculated as follows:

(6) $$\varepsilon_{c}^{p}=\varepsilon_{c}-\frac{1}{E_{c}}\left(\sigma_{c}-2\nu_{c}\sigma_{l}\right)$$

(7) $$\sigma_{l}=\frac{2E_{frp}\;t_{frp}\;\varepsilon_{l}}{D}$$

where $\;\varepsilon_{l}$ is the lateral strain of the FRP wrap and is manually selected from 0 to the ultimate tensile strain. In Abaqus^®^, material properties can be made dependent on the so-called “solution-dependent field variables” (SDFV) using the user-defined subroutine USDFLD. The SDFV is a field variable that varies throughout the solution process. In this study, the plastic dilation angle relationship was calculated using the stated equations and entered into the model as tabular data through the SDFV option.

Finally, to define the DP hardening law in the program, the relationship between axial plastic strain ($\varepsilon_{c}^{p}$) and yield stress should be defined. The values of concrete axial strain ($\varepsilon_{c}$) and axial stress ($\sigma_{c}$) are needed to calculate these parameters at each displacement increment. In this study, $\varepsilon_{c}$ and $\sigma_{c}$ were obtained using the analysis-oriented stress–strain model for FRP-confined concrete proposed by Jiang and Teng [43].

## 3. Validation of the FEM

**Figure 2:**
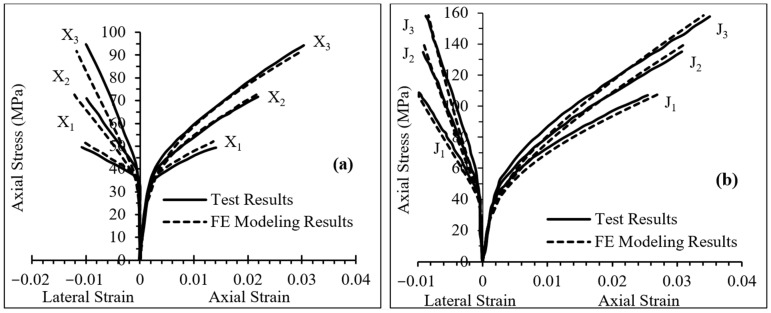

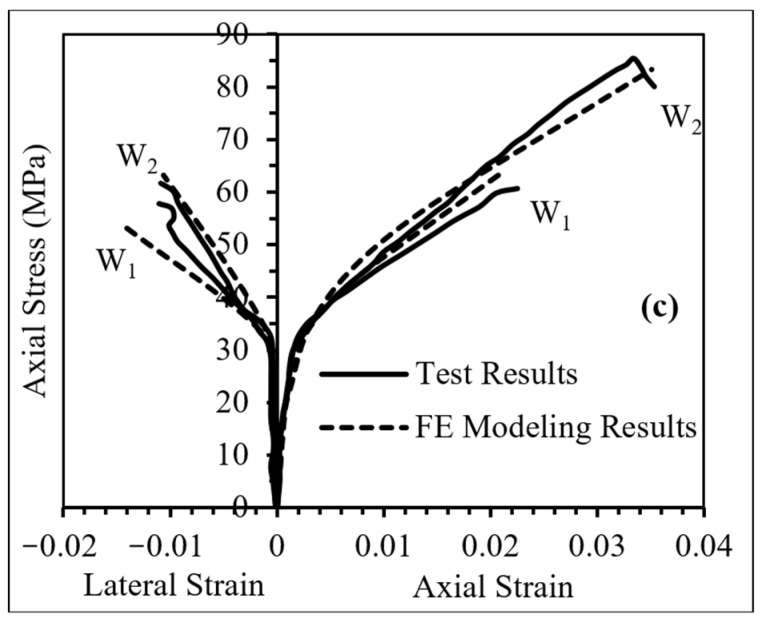
Comparison of FE modeling and experimental test results: (**a**) Specimens X1, X2 and X3, (**b**) Specimens J1, J2 and J3 and (**c**) Specimens W1 and W2.

Firstly, to select the optimum mesh size, a sensitivity analysis was performed. Mesh sizes of 10, 20 and 30 mm were considered, and the numerical results were compared to existing experimental data. All mesh sizes led to good agreement between the numerical and experimental results, and noticeable improvements were not observed when using a finer (10 mm) mesh size. Therefore, to reduce the computation time and have uniform stress contours, the optimum mesh size of 20 mm was chosen. In the next step, the accuracy of the proposed FE model was validated by numerical simulation of existing experimental results reported in the literature [39,43,50]. Table 1 illustrates the comparison between the experimental results and numerical simulation of eight independent specimens with various concrete and FRP wrap properties. These different specimens, tested in three different studies, were selected to further ensure the validity of the current FE model. It should be noted that in Table 1, $\varepsilon_{cu}$ and $f_{cu}^{\prime }$ are ultimate axial strain and ultimate axial stress of concrete confined with FRP, respectively. As shown in Figure 2, the FEA results are in strong agreement with the experimental data. The average errors were 2.9% and 4.6% for ultimate axial stress and strain, respectively, as shown in Table 1. Consequently, the FE modeling in the present study was considered valid and the parametric study could be conducted.

## 4. Parametric Study

In this study, the validated FE model was implemented to assess the compressive characteristics of FRP-confined concrete columns, with deviation in fiber orientation from the hoop direction as the primary parameter. As mentioned before, unconfined concrete strength, FRP modulus of elasticity in hoop (0°) direction and thickness of FRP wrap were also considered as variables. The combined effects of these variables with deviation in fiber orientation were investigated. The geometry and boundary conditions of the models were kept constant during the study. Important compressive characteristics investigated in this study were stress–strain behavior, ultimate axial stress and strain and total dissipated energy. Details of parametric study models are presented in the following sections. Comprehensive numerical results were shown in Appendix A.

### 4.1. Investigated Parameters

As previously mentioned, following parameters were considered: deviation in fiber orientation with respect to hoop direction (θ), unconfined concrete compressive strength ($f_{co}^{\prime }$), FRP modulus of elasticity in hoop direction ($E_{frp}$) and FRP wrap thickness ($t_{frp}$). In order to systematically investigate the effects of deviation in fiber orientation, the θ values selected were 0°, 2°, 5°, 8°, 10° and 15° with respect to hoop direction. The values of the other three parameters are shown in Table 2, along with their assigned notations. The selected practical values covered a widespread range of material properties were used in the field. All significant combinations of these parameters have been considered and 108 specimens were modeled and analyzed.

Based on Table 2, specimen C1E1T1 means a concrete column with $f_{co}^{\prime }=20\;\mathrm{MPa}$ which was confined with the FRP wrap having $E_{frp}=50\;\mathrm{GPa}$ and $t_{frp}=0.2\;\mathrm{mm}$, for example. The mechanical properties of the FRP composites used in parametric studies are given in Table 3. It should be noted that these properties were based on two commercially available GFRP ($E_{frp}=50\;\mathrm{GPa}$) and CFRP ($E_{frp}=200\;\mathrm{GPa}$) wraps. The ultimate tensile hoop strain of the FRP wrap $\varepsilon^{*}{}_{frp}$ was obtained from tensile tests of samples.

### 4.2. Modeling Procedure of Concrete Confined using FRP with Inclined Fibers

**Figure 3:**
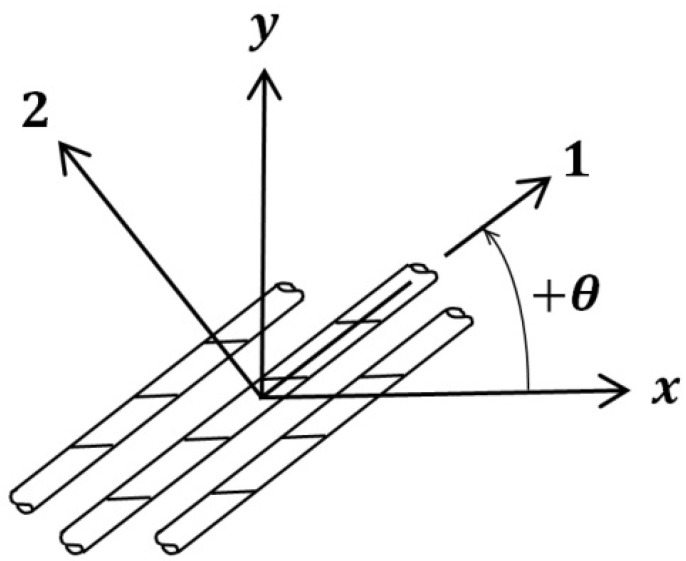
Illustration of principal axes (1, 2) and general axes (x-hoop, y-longitudinal) of a unidirectional FRP layer.

The mechanical properties of FRP composites in principal directions (1 and 2) are given in Table 3. As shown in Figure 3, when fibers had an angle θ with respect to hoop direction, the projected value of the FRP modulus of elasticity in hoop direction ($E_{x}$) decreased. Therefore, the DP parameters for confined concrete and tensile properties of FRP wrap should have been modified and recalculated accordingly [43,52] by using Equation (8):

(8) $$\frac{1}{E_{x}}=\frac{1}{E_{1}}\cos^{4}\theta +\frac{1}{E_{2}}\sin^{4}\theta +\left(\frac{1}{G_{12}}-2\frac{\nu_{12}}{E_{1}}\right)\sin^{2}\theta \cos^{2}\theta$$

It should be noted that θ represents the fiber orientation with respect to hoop direction hereafter.

### 4.3. Confined Concrete with Fibers in Hoop Direction

In order to understand the behavior of FRP-confined concrete when using fibers in hoop direction, a total of 18 configurations were modeled and the results are tabulated in Table 4. The parameter $K_{l}$ is the lateral stiffness of FRP wrap, and could be defined using Equation (9):

(9) $$K_{l}=\frac{2E_{\mathrm{frp}}t_{\mathrm{frp}}}{D}$$

In Table 4, relations $f_{cu}^{\prime }/f_{co}^{\prime }$ and $\varepsilon_{cu}^{\prime }/\varepsilon_{co}^{\prime }$ show the strength and strain improvement ratios of FRP-confined concrete, respectively. These two non-dimensional parameters were defined to facilitate the comparison between the results. $E_{t}$ was the total dissipated energy of specimens, and was defined as the product of the area underneath the axial stress–strain diagram and the volume of specimens. These three parameters defined the confinement effectiveness of FRP wraps in this study.

As observed in Table 4, the higher the $E_{frp}$ and $t_{frp}$, the more effective the confinement of the column. However, the rate of the improvement in $f_{cu}^{\prime }/f_{co}^{\prime }$, $\varepsilon_{cu}^{\prime }/\varepsilon_{co}^{\prime }$ and $E_{t}$ were more sensitive to $t_{frp}$ than to $E_{frp}$, especially for the strain improvement ratio. The increase in the aforementioned parameters was greater in specimens confined with lower $E_{frp}$, due to the larger ultimate hoop tensile strain, as shown by previous studies [40]. Table 4 shows that an increase in the $f_{co}^{\prime }$ resulted in the decrease in the above parameters and, thus, the confinement effectiveness. When concrete had a lower compressive capacity, the concrete core underwent larger dilations and, thus, would be subjected to greater radial stresses from the FRP wrap, meaning more effective confinement and higher values of $f_{cu}^{\prime }/f_{co}^{\prime }$, $\varepsilon_{cu}^{\prime }/\varepsilon_{co}^{\prime }$ and $E_{t}$.

### 4.4. Effect of Fiber Orientation on FRP Modulus of Elasticity in Hoop Direction ($E_{x}$)

**Figure 4:**
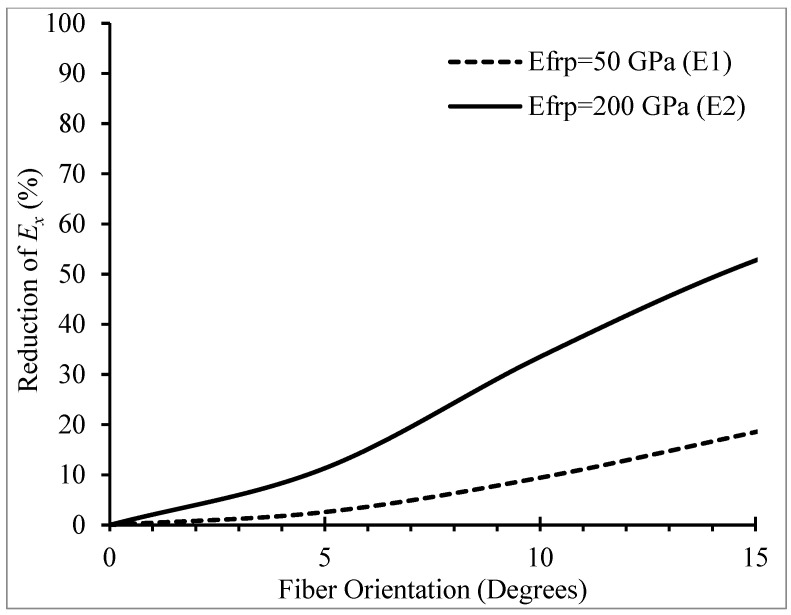
Effect of fiber orientation on FRP hoop modulus of elasticity ($E_{x}$).

As explained previously, $E_{x}$ is a function of θ and the DP parameters for confined concrete should have been modified for the new $E_{x}$ values. Therefore, it was important to explore the effect of deviation in θ on $E_{x}$ for the two FRP wraps used in this study (E1 and E2). Figure 4 shows the changes in $E_{x}$, obtained from Equation (8), for θ varying between 0° and 15°.

Figure 4 displays that the reduction in $E_{x}$ for different values of θ was more significant for the E2 wrap. It could be shown that this higher reduction was more associated with the values of in-plane shear modulus ($G_{12}$) than to the transverse modulus of elasticity (*E_2_*) of the wrap. Additionally, for 5° deviation in θ from the hoop direction, the reduction in $E_{x}$ was 2.6% and 11.3% in E1 and E2 wraps, respectively. This observation confirmed the previous concerns regarding deviation in θ, as the confinement effectiveness of the FRP wrap was highly influenced by $E_{x}$.

### 4.5. Effect of Fiber Orientation on Axial Stress–Axial and Lateral Strain Responses

**Figure 5:**
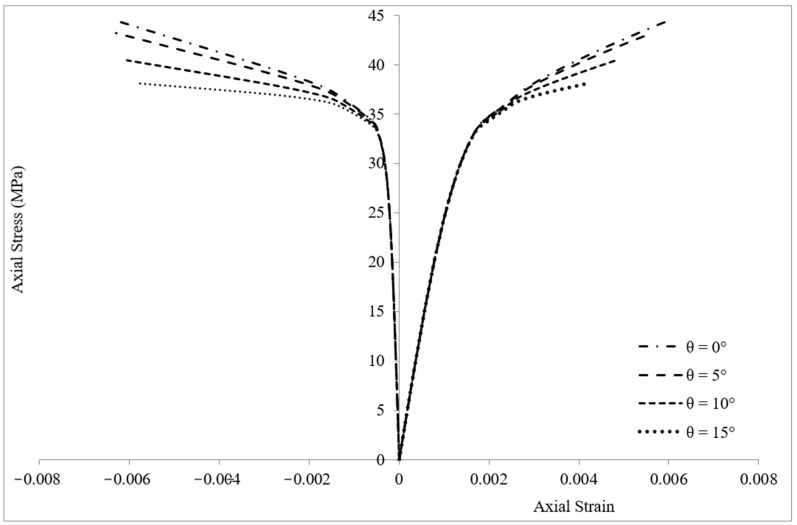
Variation of axial stress–axial and lateral strain of specimen C2E2T1 for different fiber orientations.

Figure 5 shows the variation of axial stress–axial and lateral strain of specimen C1E1T1 with θ. It could be shown that the lateral strain of concrete was equal to hoop tensile strain in the FRP wrap. Therefore, the lateral strain values in Figure 5 were equal to the data that could be obtained from a strain gauge installed on the wrap along the hoop direction.

In Figure 5, we can observe that the initial segments of the stress–strain curves were fairly linear and similar for all fiber orientations. The linear behavior lasted until axial stress got close to the unconfined concrete strength, $f_{co}^{\prime }$ (35 MPa for this specimen). From this point on, the effect of the FRP confinement appeared and the variation of the response with different fiber orientations could be distinguished. The stress–strain response featured a monotonically ascending bi-linear curve with ascending second branches (up to 15° in this study). This indicated an effective confinement, as both the compressive strength and the ultimate axial strain were significantly increased, and the column exhibited a promising ductile behavior. The descent of the second branches was caused by reduction in confinement effectiveness, since the axial stiffness of the FRP wrap was a function of $E_{x}$, and it decreased when θ increased. It could be concluded that with deviation in θ, the compressive behavior of FRP-confined concrete columns transforms from a ductile behavior to an undesired brittle behavior.

### 4.6. Variation of Concrete Axial Stress versus FRP Strain in Fiber Direction

**Figure 6:**
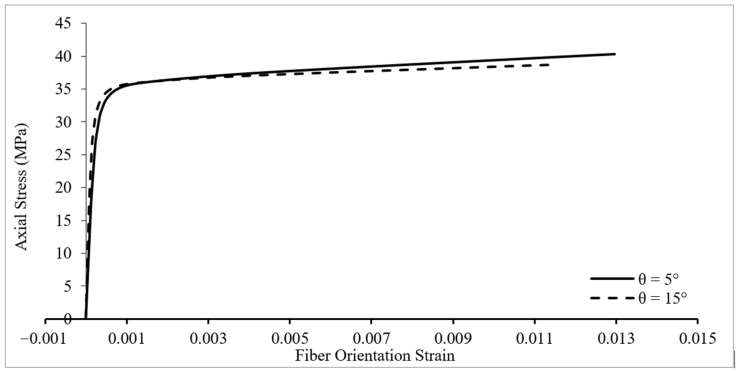
Variation of axial stress–fiber orientation strain of specimen C2E1T1 for different fiber orientations.

The lateral strain of concrete in Figure 5 was obtained assuming it was equal to the tensile strain of FRP wrap in hoop direction (direction x in Figure 3). As explained previously, the strain of FRP wrap obtained from FEA were in an x-y coordinate system, and could be easily transformed into a 1-2 coordinate system for inclined fibers. The strain of FRP wrap along axis 1 (i.e., along fiber direction) was called fiber orientation strain. Hence, fiber orientation strain was the value that a strain gauge installed along the orientation of fiber could measure. The relationship between axial stress and fiber orientation strain of specimens could be developed for different fiber angles. The response for specimen C2E1T1 is presented in Figure 6. Similar behavior was observed in all specimens.

As the fiber angle increased, the fiber orientation strain decreased remarkably and differed noticeably from the ultimate hoop tensile strain of the FRP wrap. A closer look at the trend of change in the response from 5° to 15° reveals that for higher θ values, the fiber orientation strain would become negative at the initial stages of loading (first branch of the response). This would be due to fibers aligning partially in the axial direction at higher θ values becoming subjected to compressive stresses. These observations indicated that the effectiveness of FRP confinement was at its highest when fibers were aligned in a hoop direction.

### 4.7. Effect of Fiber Orientation on Strength Improvement Ratio

**Figure 7:**
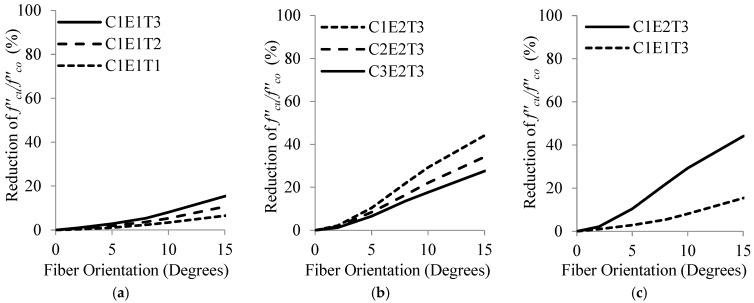
Variations of $f_{cu}^{\prime }/f_{co}^{\prime }$ versus fiber orientation for selected specimens: (**a**) for $t_{frp}$ from 0.2 to 1.0 mm, (**b**) for $f_{co}^{\prime }$ from 20 to 50 MPa and (**c**) for $E_{frp}$ of 50 and 200 GPa.

In this section, the effect of deviation in θ on compressive strength of FRP-wrapped specimens was investigated. The non-dimensional parameter $f_{cu}^{\prime }/f_{co}^{\prime }$, introduced previously, was used to compare these results with that of fibers in the hoop direction. Figure 7 presents graphical comparisons of variations of $f_{cu}^{\prime }/f_{co}^{\prime }$ with fiber orientation, along with the effect of $f_{co}^{\prime }$, $E_{frp}$ and $t_{frp}$.

It is clear from Figure 7 that $f_{cu}^{\prime }/f_{co}^{\prime }$ decreased substantially as θ increased. It could be observed that this reduction was positively associated with the $E_{frp}$ and $t_{frp}$, and was negatively associated with the $f_{co}^{\prime }$. In other words, specimens which exhibited higher enhancement in $f_{cu}^{\prime }/f_{co}^{\prime }$ with hoop fibers (i.e., specimens with higher $E_{frp}$ and $t_{frp}$, and lower $f_{co}^{\prime }$), showed higher reduction in $f_{cu}^{\prime }/f_{co}^{\prime }$ with deviation in θ. A careful comparison between Figure 7 and Figure 4 reveals that in specimens wrapped with a specific $E_{frp}$, the trend of reduction in $f_{cu}^{\prime }/f_{co}^{\prime }$ with θ was similar to the trend of reduction in $E_{x}$ of that wrap.

Since the primary objective of rehabilitation projects was enhancing the strength and load-carrying capacity, it was beneficial to study the variations of $f_{cu}^{\prime }/f_{co}^{\prime }$ with deviation in θ in more detail. In Table 5, the minimum (Min), maximum (Max), average (Avg) and standard deviation (SD) of reductions in $f_{cu}^{\prime }/f_{co}^{\prime }$ as a function of $E_{frp}$, $t_{frp}$ and $f_{co}^{\prime }$ for fiber orientations ranging from 0° to 15° are tabulated. It should be noted that Min, Max, Avg and SD were defined by making the corresponding parameter constant and the other two parameters variable. For example, the statistical values given for the parameter T1 ($t_{frp}=0.2\;\mathrm{mm}$) consist of all the specimens having a wrap thickness of 0.2 mm, regardless of $E_{frp}$ and $f_{co}^{\prime }$.

Based on Table 5, the average reduction in $f_{cu}^{\prime }/f_{co}^{\prime }$ increased 2.5, 3.3 and 1.6 times when $t_{frp}$ increased from 0.2 to 1.0 mm, $E_{frp}$ increased from 50 to 200 GPa and $f_{co}^{\prime }$ decreased from 50 to 20 MPa, respectively. It could also be concluded that the variations of $f_{cu}^{\prime }/f_{co}^{\prime }$ due to deviation in θ were more sensitive to $E_{frp}$ than to $t_{frp}$ and $f_{co}^{\prime }$, based on the data points generated in the parametric studies.

The overall reduction in $f_{cu}^{\prime }/f_{co}^{\prime }$ for each θ (regardless of $E_{frp}$, $t_{frp}$ and $f_{co}^{\prime }$) are shown in Table 6. Hence, this reduction for 5° deviation in θ (allowable limit of deviation in θ in design codes) could now be observed. It should be noted that 95% CI and 99% CI stand for the upper limits of one-sided confidence intervals with 95% and 99% confidences, respectively.

As shown in Table 6, for 5° deviation in θ relative to hoop direction, the reduction in $f_{cu}^{\prime }/f_{co}^{\prime }$ ranged from 0.6% to 10.4% with an average of 2.4%. This reduction was less than 2.9% and 3.1% for 95% and 99% confidences, respectively. It was quite noticeable that for 10° and 15° deviation in θ, the average reduction in $f_{cu}^{\prime }/f_{co}^{\prime }$ escalated from 2.4% to 10.0% and 16.4%, respectively. This observation further emphasizes the importance of limiting the deviations in fiber orientation.

Table 6 presents an investigation of the reduction in $f_{cu}^{\prime }/f_{co}^{\prime }$ for different deviations in fiber orientation (design codes point of view, 5° deviation in θ). This could be looked at from the other point of view; calculating the allowable deviation in fiber orientation for selected acceptable reductions in $f_{cu}^{\prime }/f_{co}^{\prime }$, as tabulated in Table 7.

**Figure 8:**
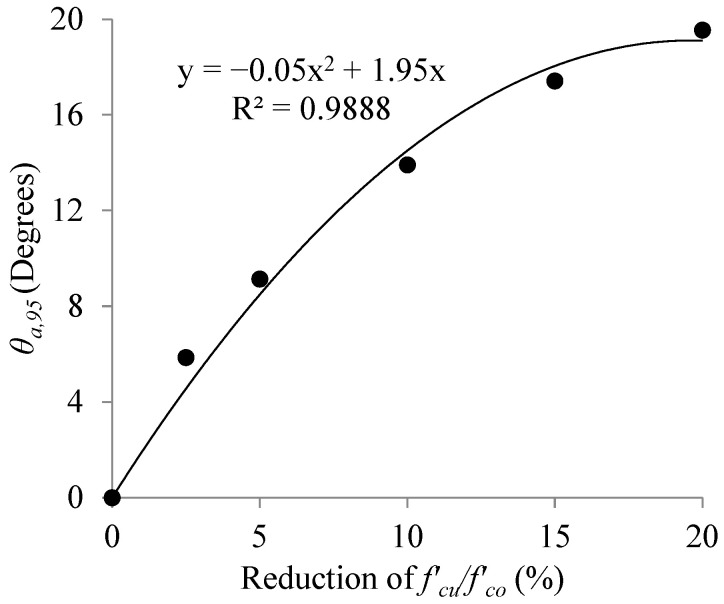
Variations of $\theta_{a,95}$ with respect to reductions in $f_{cu}^{\prime }/f_{co}^{\prime }$.

Based on Table 7, it could be stated that, for example, the fiber angle which results in a 2.5% reduction in $f_{cu}^{\prime }/f_{co}^{\prime }$, should be less than 6° with a 95% confidence. In other words, the allowable limit of deviation in θ with 95% confidence, $\theta_{a,95}$, for a 2.5% reduction in $f_{cu}^{\prime }/f_{co}^{\prime }$ is 6°. Variation of $\theta_{a,95}$ with respect to reduction in $f_{cu}^{\prime }/f_{co}^{\prime }$ is illustrated in Figure 8.

As shown in Figure 8, the best regression that fitted the two parameters was a second-order polynomial with R^2^ = 0.988. The equation obtained from regression analysis gives the value of $\theta_{a,95}$ for any given reduction in $f_{cu}^{\prime }/f_{co}^{\prime }$ up to 20%.

Table 7 displays the values of $\theta_{a}$, regardless of other parameters ($E_{frp}$, $t_{frp}$ and $f_{co}^{\prime }$). In order to provide more detailed comparisons, Table 8, Table 9 and Table 10 were developed to investigate the effect of each parameter separately.

Based on Table 8, the $\theta_{a}$ values for the E2 wrap were lower than E1. It should be noted that the E1 and E2 wraps were commercially available GFRP and CFRP composites, respectively. The CFRP wraps were more sensitive to deviation in θ than GFRP wraps because of having lower $\theta_{a}$ values. In other words, a lower deviation in fiber orientation caused the same reduction in $f_{cu}^{\prime }/f_{co}^{\prime }$ in specimens wrapped with CFRP. This could be regarded as more reduction in hoop modulus of elasticity ($E_{x}$) in the E2 wrap compared to E1 (Figure 4).

It is shown in Table 9 that as the thickness of the FRP wrap increases, the $\theta_{a}$ values decrease. In other words, the reduction in $f_{cu}^{\prime }/f_{co}^{\prime }$ with deviation in θ was higher as the wraps’ thickness increased (Figure 7 and Table 5), and the $\theta_{a}$ reduced consequently.

Based on Table 10, the lowest value of $\theta_{a}$ corresponded to $f_{co}^{\prime }=20\;\mathrm{MPa}$. This was because the reduction in $f_{cu}^{\prime }/f_{co}^{\prime }$ with deviation in θ was greater for lower values of $f_{co}^{\prime }$ (Figure 7 and Table 5). As a result, $\theta_{a}$ would be lower for smaller values of $f_{co}^{\prime }$.

### 4.8. Effect of Fiber Orientation on Strain Improvement Ratio and Total Dissipated Energy

The effect of deviation in θ on the strain enhancement ratio, $\varepsilon_{cu}^{\prime }/\varepsilon_{co}^{\prime }$, and total dissipated energy, $E_{t}$, of FRP-wrapped concrete specimens was investigated in this section. Both parameters were previously introduced. It was necessary to state that the variations of these parameters with θ and the reduction trends were very similar to that of $f_{cu}^{\prime }/f_{co}^{\prime }$, with differences in values only. Therefore, in order to avoid duplication, only the tabulated results and corresponding diagrams are reported in this section, and for discussion, readers could refer to the previous section.

**Figure 9:**
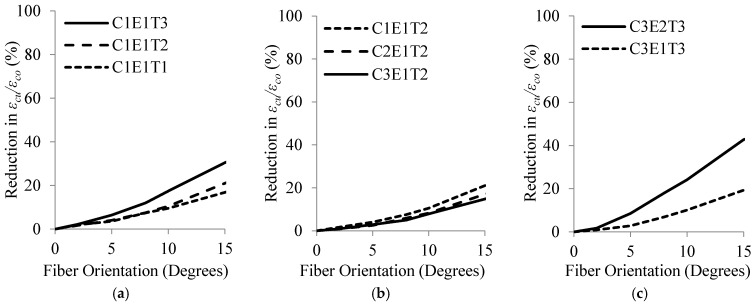
Variations of $\varepsilon_{cu}^{\prime }/\varepsilon_{co}^{\prime }$ versus fiber orientation for selected specimens: (**a**) for $t_{frp}$ from 0.2 to 1.0 mm, (**b**) for $f_{co}^{\prime }$ from 20 to 50 MPa and (**c**) for $E_{frp}$ of 50 and 200 GPa.

**Figure 10:**
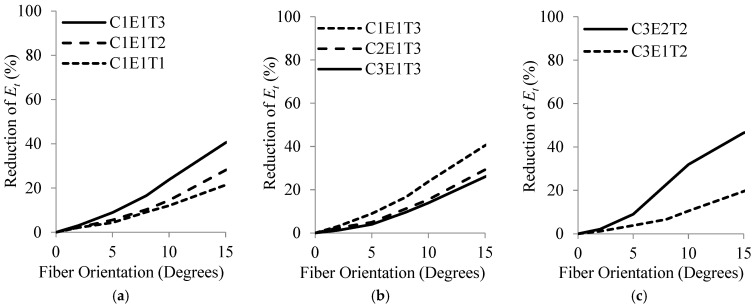
Variations of $E_{t}$ versus fiber orientation for selected specimens: (**a**) for $t_{frp}$ from 0.2 to 1.0 mm, (**b**) for $f_{co}^{\prime }$ from 20 to 50 MPa and (**c**) for $E_{frp}$ of 50 and 200 GPa.

The variations of the strain enhancement ratio, $\varepsilon_{cu}^{\prime }/\varepsilon_{co}^{\prime }$, and total dissipated energy, $E_{t}$, with respect to θ are shown in Figure 9 and Figure 10, respectively. Table 11 presents the average reductions in $\varepsilon_{cu}^{\prime }/\varepsilon_{co}^{\prime }$ and $E_{t}$ for θ ranging from 0° to 15° corresponding to different values of $E_{frp}$, $t_{frp}$ and $f_{co}^{\prime }$.

As tabulated in Table 11, the average reduction in both parameters increased as $E_{frp}$ and $t_{frp}$ increased and $f_{co}^{\prime }$ decreased. Additionally, the variation of the parameters was more affected by $E_{frp}$ than by $t_{frp}$ and $f_{co}^{\prime }$, similar to what was previously observed with $f_{cu}^{\prime }/f_{co}^{\prime }$.

Table 12 shows the overall reduction in $\varepsilon_{cu}^{\prime }/\varepsilon_{co}^{\prime }$ and $E_{t}$ for different fiber orientations. The reduction in $\varepsilon_{cu}^{\prime }/\varepsilon_{co}^{\prime }$ and $E_{t}$ for 5° of deviation in θ was less than 3.4% and 5.5% with 95% confidence, respectively. It was rather noteworthy that for 10° of deviation in θ, the average reduction in $\varepsilon_{cu}^{\prime }/\varepsilon_{co}^{\prime }$ and $E_{t}$ increased significantly about six- and fivefold, respectively. Based on this observation and considering the crucial role of ductility and energy dissipation capacity in structural concrete elements, especially in earthquake-prone regions, it was important to take into account the adverse effects of deviation in fiber orientation.

**Figure 11:**
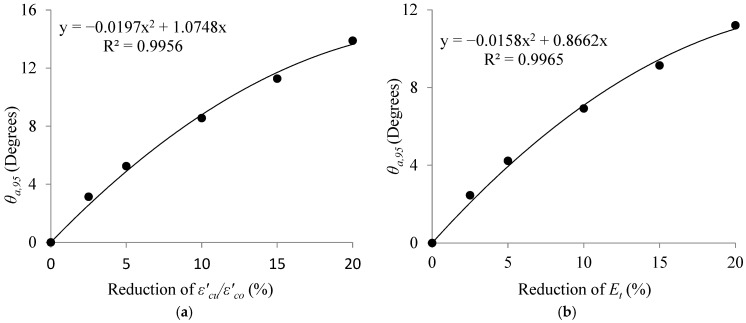
Variations of $\theta_{a,95}$ with respect to reductions of (**a**) $f_{cu}^{\prime }/f_{co}^{\prime }$ and (**b**) $E_{t}$.

Finally, Table 13 provides the calculated values of $\theta_{a,95}$ and $\theta_{a,99}$ for different acceptable reductions in $\varepsilon_{cu}^{\prime }/\varepsilon_{co}^{\prime }$ and $E_{t}$. As tabulated in Table 13, the allowable limits of deviation in θ with 95% confidence, $\theta_{a,95}$, for a 2.5% reduction in $\varepsilon_{cu}^{\prime }/\varepsilon_{co}^{\prime }$ and $E_{t}$ were 3° and 2°, respectively. Figure 11a,b shows the variation of $\theta_{a,95}$ with respect to reduction in $\varepsilon_{cu}^{\prime }/\varepsilon_{co}^{\prime }$ and $E_{t}$, respectively. The best regression fitting the two parameters was similarly a second-order polynomial with R^2^ = 0.99.

### 4.9. Comparison between Variations of Strength Improvement Ratio, Strain Improvement Ratio and Total Dissipated Energy with Deviation in Fiber Orientation

**Figure 12:**
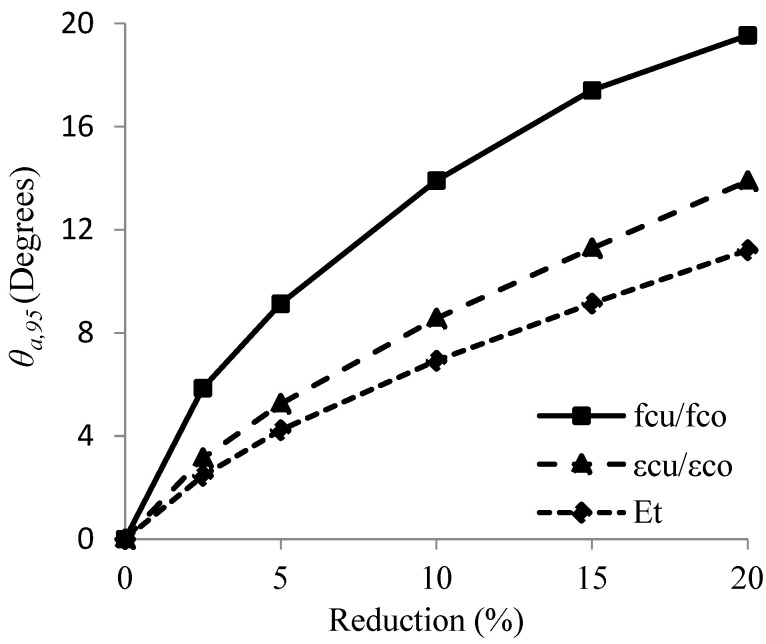
Comparison between allowable limits of deviation in fiber orientations ($\theta_{a,95}$) for each compressive characteristic.

In this part, the results presented in previous sections will be compared. The crucial issue was to find out which compressive characteristic among strength improvement ratio, strain improvement ratio or total dissipated energy were the most sensitive to deviations in θ. In Figure 12, the obtained values of $\theta_{a,95}$ (from Table 7 and Table 13) for each of the three characteristics are presented and compared graphically.

It could be observed from that the $\theta_{a,95}$ values corresponding to reduction in the strength improvement ratio were greater than that of the strain improvement ratio and total dissipated energy. This indicates that the strength improvement ratio was less sensitive to deviations in θ than the strain improvement ratio and the total dissipated energy. It also signifies that the total dissipated energy decreased the most due to deviations in θ.

### 4.10. Potential Application of the Results

The findings confirmed the acceptable deviation from the intended direction of fiber alignment (5°) given by the current ACI 440.2 design guidelines. Based on the results, simple stiffness or deformation models that take into account the fiber orientation as a variable could be developed, so designers could quickly generate stiffness or deformation values based on the prescribed material system.

## 5. Conclusions

This paper reported the results of a numerical investigation into the effects of small deviations in fiber orientation from the hoop direction (θ) as the primary variable on the compressive characteristics of FRP-confined concrete columns using the FEA. Three significant parameters affecting the confinement effectiveness, namely unconfined concrete strength ($f_{co}^{\prime }$), FRP modulus of elasticity in hoop direction ($E_{frp}$) and FRP wrap thickness ($t_{frp}$) were considered as well. θ values considered were 0°, 2°, 5°, 8°, 10° and 15° with respect to hoop direction. The $f_{co}^{\prime }$ values were 20, 35 and 50 MPa, the $E_{frp}$ values were 50 and 200 GPa and the $t_{frp}$ values were 0.2, 0.5 and 1.0 mm. The combination of all these parameters resulted in a total of 108 numerical specimens. The current allowable limit on the deviation in fiber orientation of 5°, as specified by the ACI 440.2R Design Guidelines, was evaluated in an attempt to provide a basis for the limit. The adverse effects of deviation in fiber orientation were investigated for the strength improvement ratio, strain improvement ratio and total dissipated energy of the specimens. The strength improvement ratio and strain improvement ratio were non-dimensional parameters defined as the ultimate axial stress and strain of FRP-confined concrete divided by the unconfined concrete compressive strength and peak strain, respectively. The total dissipated energy was defined as the product of the area underneath the axial stress–strain diagram and the volume of the specimens. The allowable limits of deviation in fiber orientation were calculated with practical confidences (95% and 99%) for a range of reductions in strength improvement ratio, strain improvement ratio and total dissipated energy. Based on the results and discussions presented in this paper, the following conclusions can be drawn:

- For 5° deviation in fiber orientation, the average reduction in strength improvement ratio, strain improvement ratio and total dissipated energy was 2.4%, 2.8% and 4.5%, respectively. These numbers confirm the acceptable deviation from the intended direction of fiber alignment (5°) given by the current ACI 440.2 Design Guidelines.
- The calculated allowable limit of deviation in fiber orientation for a 2.5% reduction in strength improvement ratio, strain improvement ratio, and total dissipated energy was 6°, 3° and 2°, respectively, with a 95% confidence. In other words, with 6° deviations in fiber orientation, the strength improvement ratio would reduce by 2.5%, with a 95% confidence. Or, to limit the reduction in total dissipated energy to 2.5%, the deviation in fiber orientation should be less than 2°, with a 95% confidence.
- The total dissipated energy reduced the most with deviation in fiber orientation, followed by the strain improvement ratio and strength improvement ratio.
- The adverse effects of deviation in fiber orientation were positively associated with $E_{frp}$ and $t_{frp}$, and negatively associated with $f_{co}^{\prime }$. In other words, the effectiveness of FRP confinement reduced the most in specimens with higher FRP modulus of elasticity and wrap thickness, and lower concrete compressive strength.
- For the numerical specimens analyzed, the CFRP wrap was more sensitive than GFRP to deviation in fiber orientation, considering the strength improvement ratio.
- The reduction in the strength improvement ratio, strain improvement ratio, and total dissipated energy followed a similar trend to that of the FRP hoop modulus of elasticity ($E_{x}$) with deviation in fiber orientation.
- The reduction in the strength improvement ratio, strain improvement ratio and total dissipated energy with deviation in fiber orientation was more sensitive to the FRP modulus of elasticity than the FRP wrap thickness or concrete compressive strength.

## Figures and Tables

**Table 1:** Comparison of FE modeling and experimental test results.

ID	Source	$f_{co}^{\prime }\;(\mathrm{MPa})$	$E_{frp}\;(\mathrm{GPa})$	$t_{frp}\;(\mathrm{mm})$	$\varepsilon_{cu}\;(\%)$	$\varepsilon_{cu}\;(\mathrm{FE})\;(\%)$	Error (%)	$f_{cu}^{\prime }\;(\mathrm{MPa})$	$f_{cu}^{\prime }\;(\mathrm{FE})\;(\mathrm{MPa})$	Error (%)
X1	Xiao and Wu [42]	34	105	0.38	1.41	1.36	3.6	49.3	52.1	5.7
X2	Xiao and Wu [42]	34	105	0.76	2.18	2.21	1.4	71.8	73.5	2.4
X3	Xiao and Wu [42]	34	105	1.14	3.04	2.99	1.6	94.2	91.6	2.8
J1	Jiang and Teng [46]	38	240.7	0.68	2.53	2.69	6.3	106.9	107.3	0.4
J2	Jiang and Teng [46]	38	240.7	1.02	3.08	3.12	1.3	135.1	139.1	2.9
J3	Jiang and Teng [46]	38	240.7	1.36	3.42	3.51	2.6	158.4	157.8	0.4
W1	Wang and Wu [51]	31	230.5	0.33	2.25	2.12	5.8	60.7	63.9	5.3
W2	Wang and Wu [51]	31	230.5	0.66	3.53	3.48	1.4	80.1	83.3	4.0

**Table 2:** Considered values and notations for unconfined concrete compressive strength $f_{co}^{\prime }$, FRP modulus of elasticity $\;E_{frp}$ and FRP wrap thickness $t_{frp}$.

$f_{co}^{\prime }\;\left(\mathrm{MPa}\right)$	Notation	$E_{frp}\left(\mathrm{GPa}\right)$	Notation	$t_{frp}\;\left(\mathrm{mm}\right)$	Notation
20	C1	50	E1	0.2	T1
35	C2	200	E2	0.5	T2
50	C3			1.0	T3

**Table 3:** Mechanical properties of FRP laminates used in parametric studies.

$E_{1}\left(\mathrm{GPa}\right)$	$E_{2}\left(\mathrm{GPa}\right)$	$\nu_{12}$	$G_{12}\left(\mathrm{GPa}\right)$	$G_{13}\left(\mathrm{GPa}\right)$	$G_{23}\left(\mathrm{GPa}\right)$	$\varepsilon^{*}{}_{frp}$
50	16.67	0.25	8.33	8.33	3.21	0.025
200	13.00	0.30	10.30	10.30	9.00	0.01

**Table 4:** Parametric study results of concrete confined with fibers in hoop direction.

$E_{frp}$ (GPa)	$t_{frp}$ (mm)	$K_{l}$ (MPa)	$f_{co}^{\prime }=20\;\mathrm{MPa}$			$f_{co}^{\prime }=35\;\mathrm{MPa}$			$f_{co}^{\prime }=50\;\mathrm{MPa}$		
			$f_{cu}^{\prime }/f_{co}^{\prime }$	$\varepsilon_{cu}^{\prime }/\varepsilon_{co}^{\prime }$	$E_{t}\left(mj\right)$	$f_{cu}^{\prime }/f_{co}^{\prime }$	$\varepsilon_{cu}^{\prime }/\varepsilon_{co}^{\prime }$	$E_{t}\left(mj\right)$	$f_{cu}^{\prime }/f_{co}^{\prime }$	$\varepsilon_{cu}^{\prime }/\varepsilon_{co}^{\prime }$	$E_{t}\left(mj\right)$
50	0.2	132	1.3	4.2	1.0	1.2	2.9	1.2	1.1	2.3	1.4
50	0.5	329	1.8	7.6	2.3	1.5	4.6	2.4	1.3	3.6	2.6
50	1.0	658	2.7	15.3	6.6	1.9	7.6	4.9	1.7	5.5	4.8
200	0.2	526	1.5	4.0	1.0	1.3	2.7	1.2	1.2	2.2	1.4
200	0.5	1316	2.3	7.2	2.6	1.8	5.0	2.9	1.5	3.5	2.7
200	1.0	2632	3.9	11.9	6.6	2.5	7.2	5.6	2.1	5.5	5.5

**Table 5:** Reduction in $f_{cu}^{\prime }/f_{co}^{\prime }$ due to deviation in fiber orientation for different values of $E_{frp}$, $t_{frp}$ and $f_{co}^{\prime }$.

Parameter	$\mathrm{Reduction}\;\mathrm{in}\;f_{cu}^{\prime }/f_{co}^{\prime }$ (%) for θ from 0° to 15°			
	Min	Max	Avg	SD
$t_{frp}=0.2\;\mathrm{mm}$ (T1)	0.1	17.5	4.4	4.1
$t_{frp}=0.5\;\mathrm{mm}$ (T2)	0.2	30.9	7.5	7.5
$t_{frp}=1.0\;\mathrm{mm}$ (T3)	0.4	44.1	11.1	11.0
$E_{frp}=50\;\mathrm{GPa}$ (E1)	0.1	15.4	3.5	3.4
$E_{frp}=200\;\mathrm{GPa}$ (E2)	0.7	44.1	11.8	10.0
$f_{co}^{\prime }=20\;\mathrm{MPa}$ (C1)	0.3	50.2	13.5	13.6
$f_{co}^{\prime }=35\;\mathrm{MPa}$ (C2)	0.3	38.5	10.1	10.2
$f_{co}^{\prime }=50\;\mathrm{MPa}$ (C3)	0.1	32.4	8.4	8.5

**Table 6:** Overall reduction in $f_{cu}^{\prime }/f_{co}^{\prime }$ for different deviations in fiber orientation.

Deviation in Fiber Orientation (Degrees)	$\mathrm{Reduction}\;\mathrm{in}\;f_{cu}^{\prime }/f_{co}^{\prime }\;(\%)$					
	Min	Max	Avg	SD	95% CI	99% CI
2	0.1	3.5	1.0	0.8	1.2	1.2
5	0.6	10.4	2.4	2.9	2.9	3.1
8	1.4	21.8	7.3	5.8	8.4	8.8
10	2.1	29.2	10.0	7.6	11.4	11.9
15	4.2	44.1	16.4	11.0	18.4	19.2

**Table 7:** Fiber orientations for different reductions in $f_{cu}^{\prime }/f_{co}^{\prime }$.

$\mathrm{Reduction}\;\mathrm{in}\;f_{cu}^{\prime }/f_{co}^{\prime }\;(\%)$	Deviation in Fiber Orientation (Degrees)					
	Min	Max	Avg	SD	$95\%\;\mathrm{CI}\;(\theta_{a,95})$	$99\%\;\mathrm{CI}\;(\theta_{a,99})$
2.5	2	11	5	3	6	6
5	3	17	8	4	9	9
10	5	26	13	6	14	14
15	6	29	16	7	17	18
20	8	30	18	7	20	20

**Table 8:** Fiber orientations corresponding to different reductions in $f_{cu}^{\prime }/f_{co}^{\prime }$ for each value of $E_{frp}$.

$\mathrm{Reduction}\;\mathrm{in}\;f_{cu}^{\prime }/f_{co}^{\prime }\;(\%)$	Fiber Orientation (Degrees)									
	$E_{frp}=50\;\mathrm{GPa}\;(E1)$					$E_{frp}=200\;\mathrm{GPa}\;(E2)$				
	Min	Max	Avg	$\theta_{a,95}$	$\theta_{a,99}$	Min	Max	Avg	$\theta_{a,95}$	$\theta_{a,99}$
2.5	5	11	8	8	9	2	5	3	4	4
5	8	17	12	13	13	3	8	5	6	6
10	11	26	18	20	20	5	14	8	9	10
15	15	29	23	25	26	6	24	12	14	15
20	19	27	24	26	27	8	29	15	17	19

**Table 9:** Fiber orientations corresponding to different reductions in $f_{cu}^{\prime }/f_{co}^{\prime }$ for each value of $t_{frp}$.

$\mathrm{Reduction}\;\mathrm{in}\;f_{cu}^{\prime }/f_{co}^{\prime }\;(\%)$	Fiber Orientation (Degrees)														
	$t_{frp}=0.2\;\mathrm{mm}\;(T1)$					$t_{frp}=0.5\;\mathrm{mm}\;(T2)$					$t_{frp}=1\;\mathrm{mm}\;(T3)$				
	Min	Max	Avg	$\theta_{a,95}$	$\theta_{a,99}$	Min	Max	Avg	$\theta_{a,95}$	$\theta_{a,99}$	Min	Max	Avg	$\theta_{a,95}$	$\theta_{a,99}$
2.5	4	11	7	8	9	2	9	5	7	7	2	7	4	5	5
5	6	17	11	13	14	4	13	8	10	11	3	10	6	8	8
10	6	23	13	16	17	5	21	10	12	13	3	16	8	9	10
15	9	24	14	17	18	6	29	14	17	18	4	21	10	13	13
20	13	29	20	25	27	8	25	14	18	19	5	27	13	16	17

**Table 10:** Fiber orientations corresponding to different reductions in $f_{cu}^{\prime }/f_{co}^{\prime }$ for each value of $f_{co}^{\prime }$.

$\mathrm{Reduction}\;\mathrm{in}\;f_{cu}^{\prime }/f_{co}^{\prime }\;(\%)$	Fiber Orientation (Degrees)														
	$f_{co}^{\prime }=20\;\mathrm{MPa}\;(C1)$					$f_{co}^{\prime }=35\;\mathrm{MPa}\;(C2)$					$f_{co}^{\prime }=50\;\mathrm{MPa}\;(C3)$				
	Min	Max	Avg	$\theta_{a,95}$	$\theta_{a,99}$	Min	Max	Avg	$\theta_{a,95}$	$\theta_{a,99}$	Min	Max	Avg	$\theta_{a,95}$	$\theta_{a,99}$
2.5	1	8	4	5	6	2	10	5	6	7	2	11	5	7	7
5	2	13	6	8	9	3	15	7	9	10	3	17	8	11	12
10	3	21	9	13	14	4	26	12	16	17	5	27	13	17	18
15	4	29	12	15	16	6	29	14	17	18	6	29	16	19	20
20	5	25	13	16	17	8	25	14	18	19	8	27	16	20	22

**Table 11:** Reduction in $\varepsilon_{cu}^{\prime }/\varepsilon_{co}^{\prime }$ and $E_{t}$ due to deviation in fiber orientation for different values of $E_{frp}$, $t_{frp}$ and $f_{co}^{\prime }$.

Parameter	Deviation in Fiber Orientation from 0° to 15°			
	$\mathrm{Reduction}\;\mathrm{in}\;\varepsilon_{cu}^{\prime }/\varepsilon_{co}^{\prime }\;(\%)$		$\mathrm{Reduction}\;\mathrm{in}\;E_{t}\;(\%)$	
	Avg	SD	Avg	SD
$t_{frp}=0.2\;\mathrm{mm}$ (T1)	10.7	9.0	14.1	11.7
$t_{frp}=0.5\;\mathrm{mm}$ (T2)	13.9	12.3	18.9	16.3
$t_{frp}=1.0\;\mathrm{mm}$ (T3)	15.4	13.0	22.3	18.3
$E_{frp}=50\;\mathrm{GPa}$ (E1)	7.9	6.7	10.6	8.9
$E_{frp}=200\;\mathrm{GPa}$ (E2)	18.7	13.1	26.2	17.7
$f_{co}^{\prime }=20\;\mathrm{MPa}$ (C1)	20.4	16.7	27.9	21.9
$f_{co}^{\prime }=35\;\mathrm{MPa}$ (C2)	18.1	16.1	24.1	20.3
$f_{co}^{\prime }=50\;\mathrm{MPa}$ (C3)	15.3	14.4	20.6	18.5

**Table 12:** Overall reduction in $\varepsilon_{cu}^{\prime }/\varepsilon_{co}^{\prime }$ and $E_{t}$ for different fiber orientations.

Fiber Orientation (Degrees)	$\mathrm{Reduction}\;\mathrm{in}\;\varepsilon_{cu}^{\prime }/\varepsilon_{co}^{\prime }\;(\%)$				$\mathrm{Reduction}\;\mathrm{in}\;E_{t}\;(\%)$			
	Avg	SD	95% CI	99% CI	Avg	SD	95% CI	99% CI
2	2.3	1.9	2.7	2.8	3.2	2.5	3.7	3.9
5	2.8	3.3	3.4	3.7	4.5	5.3	5.5	5.9
8	12.6	6.3	13.7	14.2	17.9	9.8	19.7	20.5
10	16.9	8.3	18.4	19.1	23.7	12.5	26.0	27.0
15	28.4	12.2	30.7	31.6	37.9	16.6	41.0	42.2

**Table 13:** Fiber orientations for different reductions in $\varepsilon_{cu}^{\prime }/\varepsilon_{co}^{\prime }$ and $E_{t}$.

Reduction (%)	Deviation in Fiber Orientation (Degrees)							
	$\varepsilon_{cu}^{\prime }/\varepsilon_{co}^{\prime }$				$E_{t}$			
	Avg	SD	$\theta_{a,95}$	$\theta_{a,99}$	Avg	SD	$\theta_{a,95}$	$\theta_{a,99}$
2.5	3	2	3	3	2	1	2	2
5	5	2	5	5	4	2	4	4
10	8	3	9	9	6	3	7	7
15	11	4	11	12	9	4	9	9
20	13	5	14	14	10	4	11	12

## Data Availability

Some or all data, models, or codes that support the findings of this study are available from the corresponding author upon reasonable request.

## Notation

The following symbols are used in this paper:

θ	Deviation in fiber orientation from the hoop direction
$f_{cu}^{\prime }$	Ultimate compressive strength of the FRP-confined concrete
$\varepsilon_{cu}^{\prime }$	Ultimate axial strain of the FRP-confined concrete
$E_{t}$	Total dissipated energy of the FRP-confined concrete
$E_{1}$	FRP modulus of elasticity in the fiber direction
$E_{2}$	FRP modulus of elasticity in the transverse direction
$\nu_{12}$	FRP Poisson’s ratio in the plane
$G_{12}$	FRP shear modulus in the plane
$E_{c}$	Concrete modulus of elasticity
$\nu_{c}$	Concrete Poisson’s ratio
$f_{co}^{\prime }$	Unconfined concrete compressive strength
$f_{cu}^{\prime }/f_{co}^{\prime }$	Strength improvement ratio
$\varepsilon_{co}$	Axial strain at the peak stress of unconfined concrete
$\varepsilon_{cu}^{\prime }/\varepsilon_{co}$	Strain improvement ratio
φ	Angle of friction in the DP plasticity model
*K*	Flow stress ratio in the DP plasticity model
β	Dilation angle in the DP plasticity model
$\varepsilon_{c}^{p}$	Axial plastic strain
ρ	Lateral stiffness ratio
$E_{x}$	FRP modulus of elasticity in hoop direction
$E_{frp}$	FRP modulus of elasticity
$t_{frp}$	FRP wrap thickness
*D*	Diameter of concrete cylinder
$\beta_{0}$	Initial slope of β
$M_{0}$	A constant equal to 157,000
$\sigma_{l}$	Lateral stress
$\;\varepsilon_{l}$	Lateral strain of the FRP wrap
$\varepsilon_{c}$	Concrete axial strain
$\sigma_{c}$	Concrete axial stress
$\varepsilon^{*}{}_{frp}$	Ultimate tensile hoop strain of the FRP wrap
$K_{l}$	Lateral stiffness of FRP wrap
$\theta_{a}$	Allowable limit of deviation in θ
$\theta_{a,95}$	Allowable limit of deviation in θ with 95% confidence
$\theta_{a,99}$	Allowable limit of deviation in θ with 99% confidence

## Appendix A. Comprehensive Numerical Results

The numerical results obtained from finite element modeling (FEM) are presented in this section. Table A1, Table A2, Table A3, Table A4 and Table A5 show the strength improvement ratio ($f_{cu}^{\prime }/f_{co}^{\prime }$), strain improvement ratio ($\varepsilon_{cu}^{\prime }/\varepsilon_{co}^{\prime }$), and total dissipated energy ($E_{t}$) of the simulated specimens and their reductions with 2°, 5°, 8°, 10° and 15° deviation in fiber orientation, respectively.

**Table A1:** FEM results for θ=2°.

	$E_{frp}$ (GPa)	$t_{frp}$ (mm)	$\frac{f_{cu}^{\prime }}{f_{co}^{\prime }}$	Reduction in $\frac{f_{cu}^{\prime }}{f_{co}^{\prime }}\;(\%)\;$	$\frac{\varepsilon_{cu}^{\prime }}{\varepsilon_{co}^{\prime }}$	Reduction in $\frac{\varepsilon_{cu}^{\prime }}{\varepsilon_{co}^{\prime }}\;(\%)\;$	$E_{t}$ (mj)	Reduction in $E_{t}$ (%)
$f_{co}^{\prime }=20\;\mathrm{MPa}$	50	0.2	1.34	0.31	4.16	1.96	0.98	2.09
	50	0.5	1.84	0.66	7.50	1.68	2.29	2.21
	50	1	2.69	0.98	14.93	2.19	6.35	3.04
	200	0.2	1.53	0.84	3.94	2.25	1.00	3.02
	200	0.5	2.28	1.40	7.10	1.88	2.52	3.01
	200	1	3.88	2.16	11.64	2.15	6.33	3.90
$f_{co}^{\prime }=35\;\mathrm{MPa}$	50	0.2	1.16	0.33	2.87	2.37	1.20	2.81
	50	0.5	1.48	0.25	4.57	0.81	2.32	1.00
	50	1	1.96	0.75	7.45	1.70	4.76	2.34
	200	0.2	1.32	1.22	2.61	4.03	1.13	5.34
	200	0.5	1.72	3.45	4.61	8.71	2.58	11.82
	200	1	2.50	1.82	7.06	2.45	5.35	3.95
$f_{co}^{\prime }=50\;\mathrm{MPa}$	50	0.2	1.07	0.08	2.34	0.02	1.41	0.05
	50	0.5	1.33	0.24	3.52	0.94	2.52	1.16
	50	1	1.68	0.35	5.44	0.91	4.74	1.20
	200	0.2	1.23	1.29	2.14	4.36	1.33	5.91
	200	0.5	1.52	0.66	3.43	1.51	2.63	2.13
	200	1	2.03	1.23	5.37	1.71	5.31	2.74

**Table A2:** FEM results for θ=5°.

	$E_{frp}$ (GPa)	$t_{frp}$ (mm)	$\frac{f_{cu}^{\prime }}{f_{co}^{\prime }}$	Reduction in $\frac{f_{cu}^{\prime }}{f_{co}^{\prime }}\;(\%)\;$	$\frac{\varepsilon_{cu}^{\prime }}{\varepsilon_{co}^{\prime }}$	Reduction in $\frac{\varepsilon_{cu}^{\prime }}{\varepsilon_{co}^{\prime }}\;(\%)\;$	$E_{t}$ (mj)	$\mathrm{Reduction}\;\mathrm{in}\;E_{t}$ (%)
$f_{co}^{\prime }=20\;\mathrm{MPa}$	50	0.2	1.33	1.06	4.10	3.55	0.96	4.32
	50	0.5	1.81	1.90	7.32	4.03	2.21	5.55
	50	1	2.64	2.83	14.28	6.41	5.96	8.90
	200	0.2	1.48	3.91	3.66	9.19	0.91	12.48
	200	0.5	2.14	7.39	6.55	9.40	2.22	14.90
	200	1	3.55	10.40	10.76	9.53	5.45	17.27
$f_{co}^{\prime }=35\;\mathrm{MPa}$	50	0.2	1.15	1.03	2.80	4.82	1.16	5.91
	50	0.5	1.47	1.02	4.49	2.51	2.27	3.30
	50	1	1.94	1.81	7.32	3.48	4.63	4.96
	200	0.2	1.29	2.97	2.50	7.85	1.07	10.61
	200	0.5	1.67	6.38	4.33	14.24	2.36	19.54
	200	1	2.33	8.38	6.50	10.20	4.66	16.50
$f_{co}^{\prime }=50\;\mathrm{MPa}$	50	0.2	1.06	0.61	2.31	1.25	1.39	1.73
	50	0.5	1.32	1.04	3.45	2.89	2.45	3.81
	50	1	1.67	1.36	5.33	2.83	4.60	3.94
	200	0.2	1.21	2.70	2.08	7.15	1.28	9.85
	200	0.5	1.48	3.15	3.26	6.23	2.44	8.98
	200	1	1.92	6.53	5.00	8.50	4.72	13.52

**Table A3:** FEM results for θ=8°.

	$E_{frp}$ (GPa)	$t_{frp}$ (mm)	$\frac{f_{cu}^{\prime }}{f_{co}^{\prime }}$	Reduction in $\frac{f_{cu}^{\prime }}{f_{co}^{\prime }}\;(\%)\;$	$\frac{\varepsilon_{cu}^{\prime }}{\varepsilon_{co}^{\prime }}$	Reduction in $\frac{\varepsilon_{cu}^{\prime }}{\varepsilon_{co}^{\prime }}\;(\%)\;$	$E_{t}$ (mj)	Reduction in $E_{t}$ (%)
$f_{co}^{\prime }=20\;\mathrm{MPa}$	50	0.2	1.31	2.36	3.93	7.41	0.91	9.12
	50	0.5	1.78	3.66	7.05	7.52	2.10	10.25
	50	1	2.57	5.36	13.42	12.03	5.46	16.56
	200	0.2	1.42	8.09	3.29	18.51	0.78	24.71
	200	0.5	1.97	14.85	5.88	18.69	1.86	28.60
	200	1	3.10	21.83	9.36	21.29	4.24	35.57
$f_{co}^{\prime }=35\;\mathrm{MPa}$	50	0.2	1.14	1.90	2.74	6.77	1.13	8.46
	50	0.5	1.45	2.49	4.34	5.88	2.16	7.86
	50	1	1.89	4.12	6.98	7.97	4.33	11.17
	200	0.2	1.26	5.85	2.32	14.51	0.96	19.50
	200	0.5	1.58	11.44	3.83	24.23	1.97	32.69
	200	1	2.13	16.45	5.82	19.66	3.86	30.71
$f_{co}^{\prime }=50\;\mathrm{MPa}$	50	0.2	1.05	1.42	2.28	2.89	1.36	3.93
	50	0.5	1.31	2.03	3.37	4.96	2.38	6.64
	50	1	1.63	3.29	5.11	6.93	4.34	9.55
	200	0.2	1.18	5.05	1.97	12.37	1.18	16.97
	200	0.5	1.41	7.80	2.91	16.45	2.07	22.77
	200	1	1.78	13.62	4.48	18.05	3.96	27.49

**Table A4:** FEM results for θ=10°.

	$E_{frp}$ (GPa)	$t_{frp}$ (mm)	$\frac{f_{cu}^{\prime }}{f_{co}^{\prime }}$	Reduction in $\frac{f_{cu}^{\prime }}{f_{co}^{\prime }}\;(\%)\;$	$\frac{\varepsilon_{cu}^{\prime }}{\varepsilon_{co}^{\prime }}$	Reduction in $\frac{\varepsilon_{cu}^{\prime }}{\varepsilon_{co}^{\prime }}\;(\%)\;$	$E_{t}$ (mj)	Reduction in $E_{t}$ (%)
$f_{co}^{\prime }=20\;\mathrm{MPa}$	50	0.2	1.30	3.38	3.84	9.54	0.88	12.02
	50	0.5	1.75	5.30	6.83	10.50	2.00	14.43
	50	1	2.49	8.08	12.60	17.40	4.99	23.76
	200	0.2	1.38	10.54	3.09	23.32	0.71	30.89
	200	0.5	1.85	20.05	5.39	25.48	1.62	37.83
	200	1	2.80	29.24	8.40	29.41	3.51	46.69
$f_{co}^{\prime }=35\;\mathrm{MPa}$	50	0.2	1.13	2.68	2.70	8.24	1.10	10.45
	50	0.5	1.43	3.62	4.23	8.23	2.09	11.02
	50	1	1.86	5.85	6.75	10.99	4.12	15.39
	200	0.2	1.23	7.73	2.22	18.34	0.90	24.56
	200	0.5	1.51	15.02	3.49	30.94	1.73	41.05
	200	1	1.98	22.09	5.31	26.69	3.33	40.32
$f_{co}^{\prime }=50\;\mathrm{MPa}$	50	0.2	1.04	2.12	2.25	4.01	1.33	5.48
	50	0.5	1.29	3.22	3.27	7.87	2.28	10.51
	50	1	1.61	4.89	4.93	10.10	4.13	13.88
	200	0.2	1.16	6.64	1.90	15.47	1.12	21.16
	200	0.5	1.36	11.10	2.66	23.55	1.83	31.91
	200	1	1.69	17.66	4.15	24.14	3.52	35.60

**Table A5:** FEM results for θ=15°.

	$E_{frp}$ (GPa)	$t_{frp}$ (mm)	$\frac{f_{cu}^{\prime }}{f_{co}^{\prime }}$	Reduction in $\frac{f_{cu}^{\prime }}{f_{co}^{\prime }}\;(\%)\;$	$\frac{\varepsilon_{cu}^{\prime }}{\varepsilon_{co}^{\prime }}$	Reduction in $\frac{\varepsilon_{cu}^{\prime }}{\varepsilon_{co}^{\prime }}\;(\%)\;$	$E_{t}$ (mj)	Reduction in $E_{t}$ (%)
$f_{co}^{\prime }=20\;\mathrm{MPa}$	50	0.2	1.26	6.48	3.53	16.84	0.79	21.34
	50	0.5	1.65	10.61	6.02	21.02	1.68	28.04
	50	1	2.30	15.37	10.61	30.46	3.90	40.43
	200	0.2	1.27	17.45	2.50	38.10	0.53	48.40
	200	0.5	1.60	30.88	4.08	43.62	1.08	58.66
	200	1	2.22	44.07	6.27	47.29	2.16	67.16
$f_{co}^{\prime }=35\;\mathrm{MPa}$	50	0.2	1.11	5.09	2.54	13.76	1.02	17.56
	50	0.5	1.38	7.48	3.83	16.99	1.82	22.40
	50	1	1.75	11.52	5.95	21.52	3.45	29.25
	200	0.2	1.16	12.70	1.93	28.98	0.74	37.92
	200	0.5	1.37	22.97	2.77	45.23	1.24	57.52
	200	1	1.68	34.12	4.14	42.82	2.25	59.55
$f_{co}^{\prime }=50\;\mathrm{MPa}$	50	0.2	1.02	4.23	2.13	9.19	1.24	12.18
	50	0.5	1.25	6.30	3.03	14.76	2.05	19.56
	50	1	1.53	9.45	4.43	19.31	3.55	25.94
	200	0.2	1.11	11.01	1.69	24.73	0.95	33.11
	200	0.5	1.27	17.25	2.25	35.44	1.44	46.50
	200	1	1.49	27.51	3.13	42.73	2.34	57.20
